# Carbon dioxide fluxes from a degraded woodland in West Africa and their responses to main environmental factors

**DOI:** 10.1186/s13021-015-0033-6

**Published:** 2015-09-17

**Authors:** Expedit Evariste Ago, Dominique Serça, Euloge Kossi Agbossou, Sylvie Galle, Marc Aubinet

**Affiliations:** 1 Axe Exchange Ecosystems-Atmosphere, Department of Biosystem Engineering (BIOSE), University of Liege, Gembloux Agro Bio Tech, 8, Avenue de la Faculté, 5030 Gembloux, Belgium; 2 Laboratoire d’Hydraulique et de Maîtrise de l’Eau, Faculté des Sciences Agronomiques (FSA), Université d’Abomey-Calavi (UAC), BP 2819, Cotonou, Benin; 3 Laboratoire d’Aérologie, UMR CNRS 5560, Université Paul Sabatier, Toulouse, France; 4 Univ. Grenoble Alpes, LTHE, 38000 Grenoble, France; 5 CNRS LTHE, 38000 Grenoble, France; 6 IRD, LTHE, 38000 Grenoble, France

**Keywords:** Eddy-covariance, Woodland, Sudanian climate, Net ecosystem exchange, Annual NEE, Benin, West Africa, Eddy-covariance, Forêt claire, Climat soudanien, Echange net de l’écosystème, NEE annuel, Bénin, Afrique de l’Ouest

## Abstract

**Background:**

In West Africa, natural ecosystems such as woodlands are the main source for energy, building poles and livestock fodder. They probably behave like net carbon sinks, but there are only few studies focusing on their carbon exchange with the atmosphere. Here, we have analyzed CO_2_ fluxes measured for 17 months by an eddy-covariance system over a degraded woodland in northern Benin. Specially, temporal evolution of the fluxes and their relationships with the main environmental factors were investigated between the seasons.

**Results:**

This study shows a clear response of CO_2_ absorption to photosynthetic photon flux density (Q_p_), but it varies according to the seasons. After a significant and long dry period, the ecosystem respiration (R) has increased immediately to the first significant rains. No clear dependency of ecosystem respiration on temperature has been observed. The degraded woodlands are probably the “carbon neutral” at the annual scale. The net ecosystem exchange (NEE) was negative during wet season and positive during dry season, and its annual accumulation was equal to +29 ± 16 g C m^−2^. The ecosystem appears to be more efficient in the morning and during the wet season than in the afternoon and during the dry season.

**Conclusions:**

This study shows diurnal and seasonal contrasted variations in the CO_2_ fluxes in relation to the alternation between dry and wet seasons. The Nangatchori site is close to the equilibrium state according to its carbon exchanges with the atmosphere. The length of the observation period was too short to justify the hypothesis about the “carbon neutrality” of the degraded woodlands at the annual scale in West Africa. Besides, the annual net ecosystem exchange depends on the intensity of disturbances due to the site management system. Further research works are needed to define a woodland management policy that might keep these ecosystems as carbon sinks.

## Résumé

**Problématique:**

En Afrique de l’Ouest, les écosystèmes naturels comme les forêts claires constituent la principale source d’énergie, de bois d’oeuvre et de fourrage pour le bétail. Ces forêts claires se comportent probablement comme de puits nets de carbone, mais très peu d’études ont porté sur les échanges de carbone de celles-ci avec l’atmosphère. Cette étude a analysé les flux de CO_2_ mesurés pendant 17 mois à l’aide d’un système d’eddy-covariance placé au dessus d’une forêt claire dégradée au nord du Bénin. De façon spécifique, l’évolution temporelle des flux de CO_2_ et leurs relations avec les principaux facteurs environnementaux ont été étudiées suivant les saisons.

**Résultats:**

Cette etude montre une réponse claire de l’absorption du CO_2_ à la densité de flux de photons photosynthétiques, mais elle est différente selon les saisons. Après une longue et significative période sèche, la respiration de l’écosystème (R) augmente immédiatement en réaction aux premières pluies significatives. Aucune dépendance claire de la respiration de l’écosystème à la température n’a été observée. Les forêts claires dégradées ouest-africaines sont probablement neutres en considérant leurs échanges de carbone à l’échelle annuelle avec l’atmosphère. L’échange net de l’écosystème (NEE) est négatif pendant la saison humide et positif durant la saison sèche, et son cumul annuel est égal + 29 ± 16 g C m^−2^. L’écosystème apparaît être plus efficient dans la matinée et en saison humide que pendant l’après-midi et en saison sèche.

**Conclusion:**

Cette étude a montré d es variations journalières et saisonnières contrastées des flux de CO_2_ en relation avec l’alternance entre les saisons sèche et humide. Le site investigué est à l’état d’équilibre en considérant ses échanges de carbone avec l’atmosphère. La durée de la période d’observation était trop courte pour justifier l’hypothèse de la neutralité des forêts claires dégradées ouest-africaines par rapport aux échanges de carbone avec l’atmosphère à l’échelle annuelle. En outre, l’échange net de l’écosystème dépend de l’intensité des perturbations dues au système de gestion du site. D’autres recherches sont nécessaires pour définir une politique de gestion des forêts claires qui contribueraient à maintenir ceux-ci comme de puits nets de carbone.

## Background

Forests occupy approximately 42 million km^2^, representing approximately 30 % of the total land surface in the tropical, boreal and temperate lands [1–3]. Worldwide, it is recognized that these ecosystems influence strongly the global carbon cycle through their exchanges with the atmosphere of the carbon dioxide, energy, water and other gases or chemical elements [2, 4]. However, the complexity and large temporal or spatial variability of the interactions between the atmosphere and forests can significantly reduce or amplify impacts of the main anthropogenic factors on the climate change. Forests provide many services such as the hydrologic cycle regulation, biodiversity protection, food provision and other products [1, 5–7]. Also, forests are recognized to sequester overall large quantities of carbon, approximately 45 % of the total terrestrial carbon stock [8]. Therefore, a better understanding of the carbon exchanges dynamics within forests and a determination of their contribution to the global carbon cycle appear important for the studies focusing on interactions between these vegetation types and the atmosphere. Forest responses to the main meteorological factors whose changes can favor or limit the vegetation development increase the importance of the scientific community [9–11]. Nemani et al. [12] underline that the vegetation growth is strongly limited by water availability over 40 % of the vegetated surface of the Earth while the temperature and radiation would respectively limit this growth only to 33 and 27 %. It appears relevant to improve the overall climate impacts on the plant growth in order to better forecast the future vegetation patterns, especially in the climate change context. The dynamics of carbon fluxes within the terrestrial ecosystems and with the atmosphere could help to define the strategies to better mitigate the impacts of the variability and change of climate [1, 4, 13].

Tropical forests cover 7–10 % of the global land area which store approximately 40–50 % of the total terrestrial vegetation carbon [1, 14], mainly through the balance between respiration and photosynthesis processes. Moreover, the vegetation growth seems to be strongly limited by the drought conditions occurring overall in tropical regions during the dry seasons [15, 16]. In recent years, the tropical forests seem to maintain at a high level their evapotranspiration rate and carbon storage in relation not only to the increase in the air temperature and atmospheric CO_2_ concentration, but also to the annual rainfall improvements at the tropical regions [9, 17–19]. They act mostly as net carbon sinks [1, 7, 19, 20]. However, the future of these ecosystems seems to be uncertain not only because of the climate change, but also because of the anthropogenic pressures in relation with the high population growth rates [1, 4, 5, 21].

Over the last decades, although they have been subject to intensive human pressures, African forests have responded to the satisfaction of the needs of the populations or several environmental challenges [1, 5, 6, 13]. In West Africa, the woodlands and forests remain very important because they are the main source for energy, building poles and livestock fodder for both the rural and urban populations. In recent years, only few studies have focused on the water and carbon exchanges between the atmosphere and woodlands or forests have been reported in West Africa, especially in Benin [22, 23], Mali [24] and Niger [25, 26]. In Benin, the woodlands are mostly located in the northern part of the country where the Nangatchori site is located and occupy almost two-thirds of the total dense forest area with a woody cover between 40 and 75 % [27]. Increasingly, these ecosystem types are deforested, disturbed and converted into agricultural areas [23, 28, 29].

In this study, we have analyzed water and CO_2_ fluxes measurements made for 17 months, from November 1, 2005 to March 31, 2007 in the framework of the international AMMA program (http://www.amma.org) over a degraded woodland.

The main objective of this paper is to analyze the dynamics of carbon exchanges over a degraded woodland site. Notably, we have considered the following questions: (1) How did the net exchange ecosystem (NEE) and its two major components, ecosystem respiration (R) and gross primary production (G_p_) respond to changes in the environmental conditions at the site? (2) What were the driving variables of these fluxes at the daily and seasonal scales and (3) Finally, was the degraded woodland a carbon sink or source at the annual scale during the eddy-covariance measurements period?

## Results and discussion

### Weather context, vegetation growth and fluxes overview

**Fig. 1:**
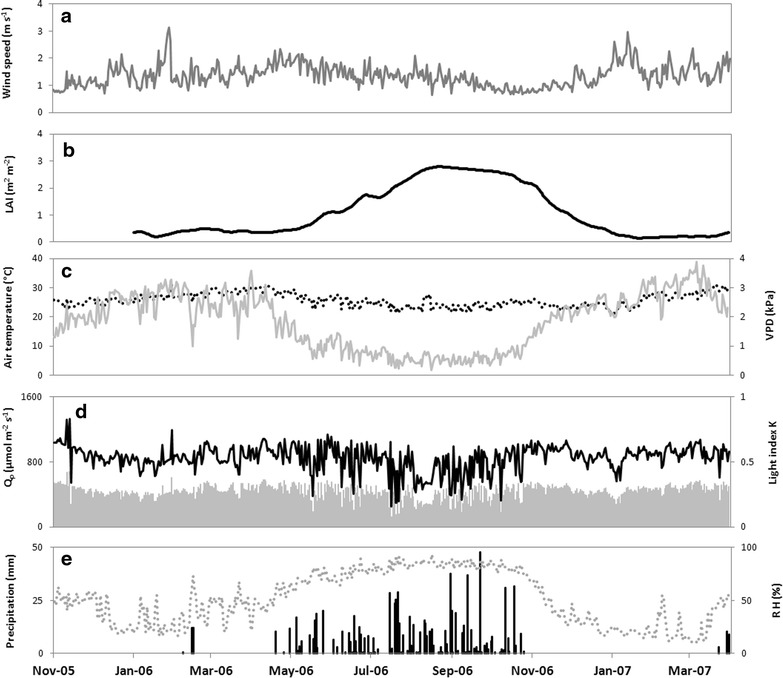
Seasonal evolution of the average daily meteorological variables from 1 November 2005 to 31 March 2007: **a** Wind speed, **b** Leaf Area Index (LAI), **c** vapour pressure deficit (VPD) (*grey continuous line*) and air temperature (*dark discontinuous line*), **d** light index (K) (*dark continuous line*) and photosynthetic photon flux density (Q_p_) (*grey histogram*), **e** precipitation (*dark histogram*) and relative humidity (RH) (*grey discontinuous line*)

In order to study the carbon fluxes dynamics over the degraded woodland, it appears useful to first describe the evolution of the key meteorological factors. Seasonal evolution of the daily average of photosynthetic photon flux density (Q_p_), light index (K), vapour pressure deficit (VPD), relative humidity (RH), air temperature, Leaf Area Index (LAI) and precipitation are given in Fig. 1. The climate at the site region is overall characterized by a succession of two main seasons, a dry one (November–April) and a wet one (May–October). This seasonality is typical of the Sudanian region, which is strongly controlled by the West African Monsoon regime, clearly highlighted through the seasonal variations of precipitation (Fig. 1e), RH (Fig. 1e) and VPD (Fig. 1c). Two transitional periods, wet-to-dry ON (October–November) and dry-to-wet AM (April–May) can be identified between the two seasons (Ago et al. submitted). During the year, the main wind direction remained South-West, except from December to January where it was North-East and dominated mostly by the Harmattan winds. The daily average speed was between 0.5 to 3.0 m s^−1^ (Fig. 1a).

There was a low seasonal variability in the daily average air temperature, which ranged between 21 and 25 °C, but reached 31 °C at the end of the dry season (Fig. 1c). The vapour pressure deficit (VPD) was lower than 0.5 kPa during the wet season and higher (2–3 kPa) during the dry one, with a maximum value of 4 kPa at the end of March (Fig. 1c).

The daily average Q_P_ varied from 335 µmol m^−2^ s^−1^ in July to 675 µmol m^−2^ s^−1^ during November (Fig. 1d). As a result, the light index K was low and more or less stable around 0.5. This was probably due to the cloudiness regime and the aerosols or dust loads brought from North-East by the Harmattan winds that reduced strongly the incoming radiation [30–32]. During the wet season, the light index K was went down to 0.2–0.3 (Fig. 1d).

Due to the Sudanian climate, most of the total precipitation is concentrated between May and October (Fig. 1e). The total rainfall during 2006 was 850 mm with 101 rainy days. This is in agreement with the regional averages for dry years from 1921 to 2009 [31, 33, 34]. The RH variability was also impacted by the Monsoon flux intrusions, South-West winds, bringing the moist from the ocean to continental surfaces in West Africa, leading to an increase in the air humidity which generally starts from February though rains have not start yet at that moment [31, 35, 36]. RH was usually high, up to approximately 90 % and low of 20–50 % during the wet and dry seasons, respectively (Fig. 1e).

LAI varied seasonally, with relatively low value during the dry season (lower value of 0.2 m^2^ m^−2^ in January) (Fig. 1b), but always significantly different from zero due to the presence of an herbaceous strata, a few crops non still harvested and sparse trees or shrubs. LAI increased continuously from January to March due to the leaves renewal for most of the woody species such as *Isoberlinia sp*, and reached a maximum value of 2.8 m^2^ m^−2^, i.e. the full development of the whole vegetation with the rain onset, between April and May months [22, 33, 37, 38].

### Footprint contributions of different vegetation types to the measured fluxes

**Fig. 2:**
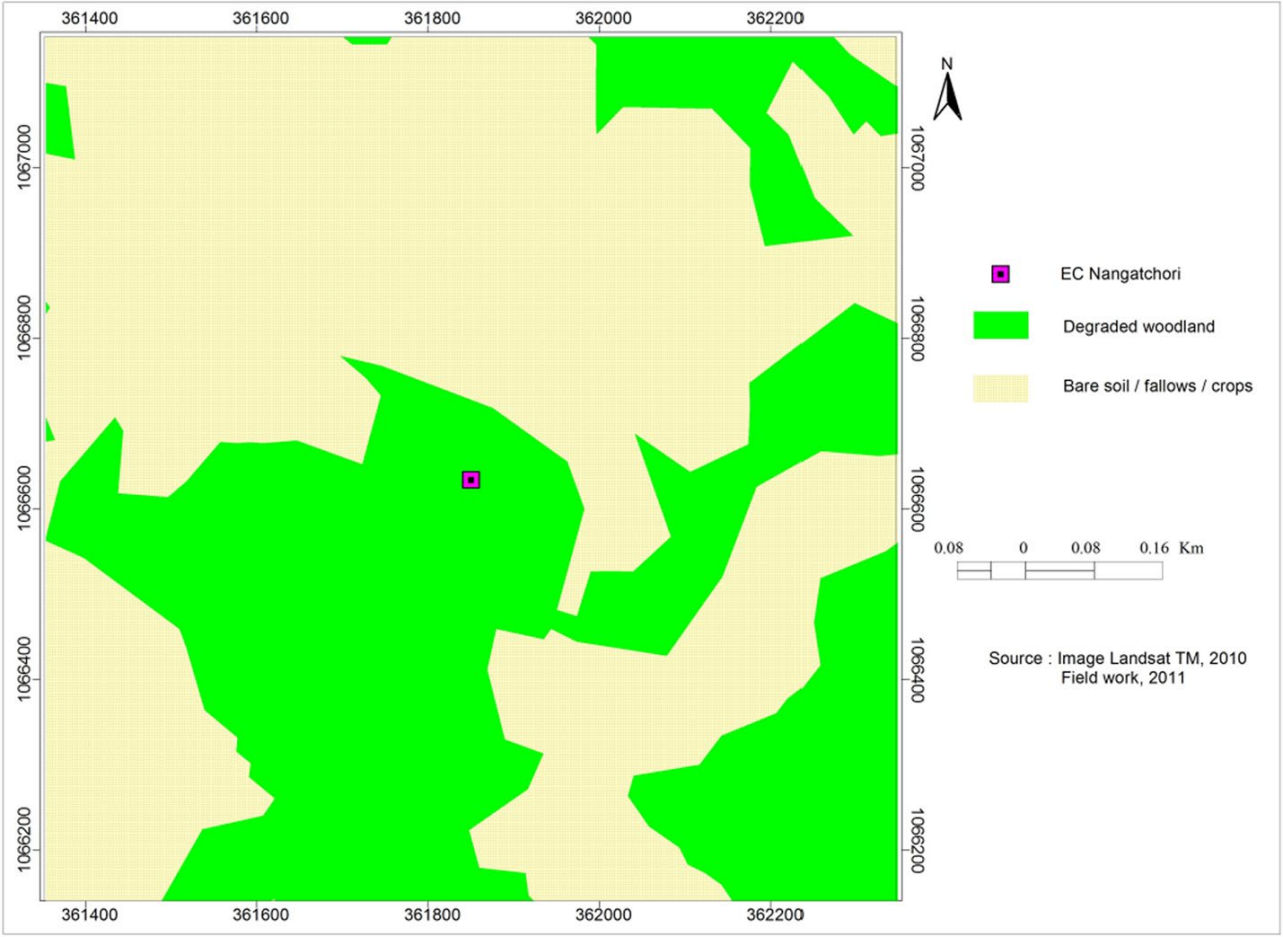
Land use distribution for a 1 km^2^ area around the flux tower

A footprint analysis was conducted to address the spatial representativeness of the measured fluxes. It reveals that each vegetation type (Fig. 2) contributed significantly to the fluxes depending on the wind occurrence from the two main directions during the year (North-East and South-West). The major contribution of the measured fluxes was emitted by the degraded woodland. Comparing the two main seasons, this contribution was more important during the wet season than the dry one. This analysis shows that the area contributing to 90 % of the measured fluxes extended up to 230 and 434 m respectively the day and night conditions.

During the dry season, the wind direction was mainly North-East and the daytime fluxes measurements were impacted primarily by the degraded woodland surroundings (71 %) and by a few perennial herbs (mostly C4 plants), bare soil and fallow (29 %). During the night, with a contribution of 64 %, the degraded woodland impacted less the measured fluxes compared to those of the day.

During the wet season, the wind primarily blew South-West when the vegetation was fully developed (Fig. 1b), especially around the tower where most of the shrubs, trees, herbs and crops covered the site area. Most of the degraded woodland being located South-West, near the tower was mainly included in the footprint areas in daytime. Therefore, measured fluxes were significantly and largely affected by the degraded woodland (74 %) followed by the cultivated areas for 26 %. In nighttime, the contribution was almost similar to 69 % of the contribution from the degraded woodland.

As a conclusion, one can say that the fluxes measured at the Nangatchori site reflected mostly those of a cultivated area and a degraded woodland respectively for the dry and wet seasons. Therefore, the contribution of trees and shrubs (mostly by C3 species) to the measured fluxes appeared all time more important than that of herbs strata or crops areas (mostly C4 species).

### Temperature response of nighttime fluxes and their relationship with relative humidity

**Fig. 3:**
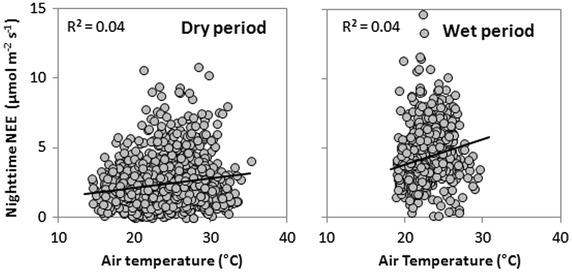
Relationship between the half-hour nighttime net ecosystem exchange (NEE) and air temperature during the wet and dry periods. Only data for u* >0.10 m s^−1^ are shown

**Fig. 4:**
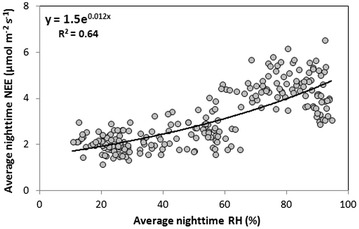
Relationship between the mean nighttime net ecosystem exchange (NEE) and mean nighttime relative humidity (RH) for the whole of period measurements (1 November 2005 to 31 March 2007). Only data for u* >0.10 m s^−1^ are shown

The plot of nighttime fluxes for unstable conditions (u* > 0.10 m s^−1^) against the half-hour air temperature measurements during both the wet and dry periods (Fig. 3) reveals no clear dependence of the nighttime ecosystem respiration on temperature. This could be due to the range of the temperature variability (daily average range <10 °C) at the Nangatchori site or masked by the response to the soil moisture [39–42]. However, a highly significant positive correlation (R^2^ = 0.63; p < 0.001) was found between the nighttime average of the ecosystem respiration and relative humidity (Fig. 4). This might suggest a significant positive correlation between nighttime averages of ecosystem respiration and soil moisture as the latter co-vary seasonally (daily scale) with RH at the site region [33]. Finally, the average of the nighttime ecosystem respiration was 1.15 ± 0.33 and 6.54 ± 2.31 µmol m^−2^ s^−1^ respectively for the dry and wet seasons.

Lack of or very little relationship was also found between nighttime fluxes and temperature for tropical forest and savanna sites [37, 43–45]. In contrast, other studies report that, for several dry west African sites, the ecosystem respiration was primarily driven by temperature [46–50] and secondly by soil moisture. For the Skukuza semi-arid South African savanna site, while Williams et al. [43] find an unclear relationship between nighttime fluxes and temperature using 52 months of data series, Kutsch et al. [51] observe only with 9 months of fluxes data a dependence on this variable. Finally, the respiration dependency with temperature seems to be influenced in the tropical regions by the temporal scale of flux measurements or combined effects of other climatic factors [45].

### Response of daytime fluxes to radiation Q_p_

**Fig. 5:**
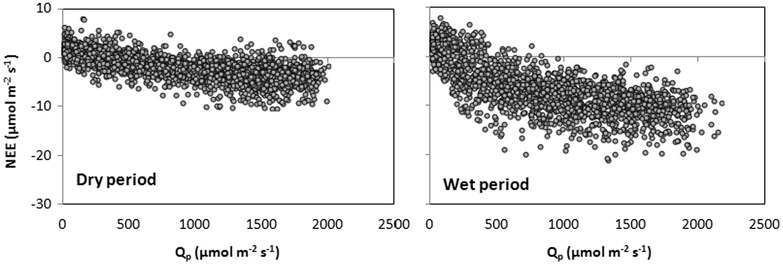
Response of the half-hour daytime net ecosystem exchange (NEE) to photosynthetic photon flux density (Q_p_) for both the wet and dry periods

It is widely recognized that light drives CO_2_ uptake at the ecosystem scale [52]. Therefore, we examined first how the daytime NEE has responded to the radiation variations, i.e. the photosynthetic photon flux density (Q_p_) changes. As, the vegetation density changes seasonall (shown by LAI seasonal variation), we have examined this relationship during the two main seasons (Fig. 5). The difference between the two seasonal variations indicates two contrasting phenological and physiological patterns. In daytime, NEE steadily increased (in absolute value) with the radiation increasing due likely to the CO_2_ absorption by the green leaves, and then saturated at high radiation (above 1000 µmol m^−2^ s^−1^). During the dry season, when the LAI value was overall lower than 0.5 m^2^ m^−2^, the light saturation was not so clear. Based on the statistics of the non linear model fitting of daytime fluxes using Eq. (1) on 15 days windows, regression coefficients indicate that the variations in NEE were explained by 50–72 % of the changes in Q_p_ confirming the dominant role played by the radiation in the CO_2_ absorption of the ecosystem. A_max_ and α varied from 1.8 ± 1.3 to 14.0 ± 1.8 µmol m^−2^ s^−1^ and 0.006 ± 0.002 to 0.040 ± 0.016 µmol µmol^−1^ respectively for dry and wet seasons.

**Fig. 6:**
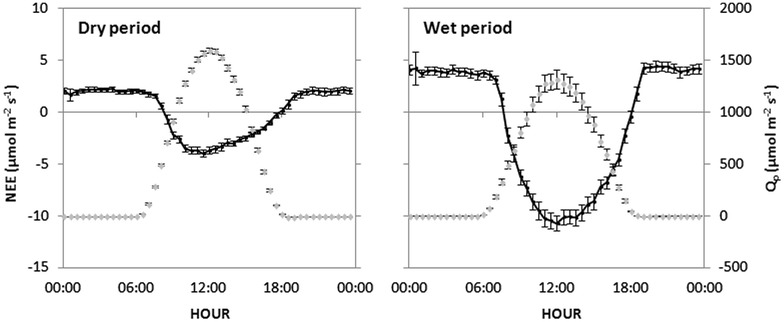
Diurnal courses of half-hour mean net ecosystem exchange (NEE) (*dark continuous line*) and photosynthetic photon flux density (Q_p_) (*grey discontinuous line* for both the dry and wet periods. *Error bars* represent 95 % confidence interval

These seasonal changes in diurnal patterns of NEE can also suggest an influence of green leaves density on respiration and photosynthesis in response to the variations in the main environmental conditions. The average diurnal courses of NEE and Q_p_ during the two main vegetation growth seasons are displayed in Fig. 6 to illustrate this assertion. In daytime, the evolution of NEE depended mainly on the Q_p_ variations and the canopy density, all two seasons showing a similar NEE curve pattern to Q_p_ (Fig. 6). Maximum values of NEE and Q_p_ were observed around noontime. During the wet season, NEE has reached −10.7 ± 0.7 µmol m^−2^ s^−1^, a value significantly higher (in absolute value) than the NEE (−4.0 ± 0.4 µmol m^−2^ s^−1^) found in the dry season. This was due to the small density of green leaves (LAI ~0.2–0.5 m^2^ m^−2^) combined to a lesser extent with higher VPD. In addition, a dissymmetry was observed in the diurnal NEE evolution more remarkably in the dry season than in the wet one (Fig. 6) suggesting a partial stomatal closure impact during the afternoon. This leads to a limitation of CO_2_ absorption by the ecosystem besides the radiation control.

This range of CO_2_ uptake at light saturation (A_max_), and quantum light efficiency (α) was well consistent with that reported in other studies for savanna and forest sites in Benin under the Sudanian climate (Ago et al. submitted). However, the corresponding A_max_ and α values were lower than those reported by Tagesson et al. [53] for a savanna grassland site in Senegal.

### Daytime water use efficiency (WUE) and evaporative fraction (EF)

**Fig. 7:**
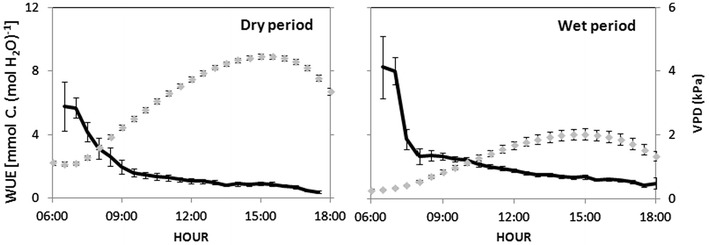
Daytime evolution of the half-hour mean water use efficiency (WUE) (*black continuous line*) and vapour pressure deficit (VPD) (*grey square*) for both the wet and dry periods. *Error bars* represent 95 % confidence interval

Daytime WUE patterns for dry and wet periods are displayed in Fig. 7. They show values ranging from 0.40 ± 0.09 to 5.79 ± 1.55 and from 0.83 ± 0.11 to 8.25 ± 1.94 mmol C (mol H_2_O)^−1^ respectively for the dry and wet periods. For both wet and dry seasons, a similar decreasing trend was found from morning to afternoon following the VPD increase. During morning, WUE was high, mainly due to the radiation increasing with low VPD. Thereafter, it declined progressively until afternoon as VPD has increased and reached its maximum value around 3.00 p.m. This suggests a partial stomatal closure impact besides the radiation control [54]. Similar daytime WUE patterns and magnitudes were reported for similar ecosystems in West Africa by several authors, especially in Benin for cultivated savanna [37] and forest sites (Ago et al. submitted); in Niger for savanna and millet crops [50, 55–57]; and in Southern Africa for savanna, woodland and forest sites [44, 58]. However, at the noontime and sunset, WUE values for our investigated site were lower than those reported for savanna and millet crops in West Africa. These differences could be explained by differences found in canopy covers of the vegetation, growth stages and species physiology [56, 59]. Indeed, savanna and millet crops canopies are generally less dense compared to those of woodland. In this latter case, larger canopies are more efficient at the intercepting rainfall leading to high subsequent evaporation from leaves and lower WUE values in the afternoon. Similar observations have been reported for Kataba forest in Zambia [58]. However, the WUE values at the Nangatchori site were consistently lower during the dry season than the wet one. This is due to variations in the vegetation growth along the year, confirmed by LAI seasonal changes (Fig. 1b).

**Fig. 8:**
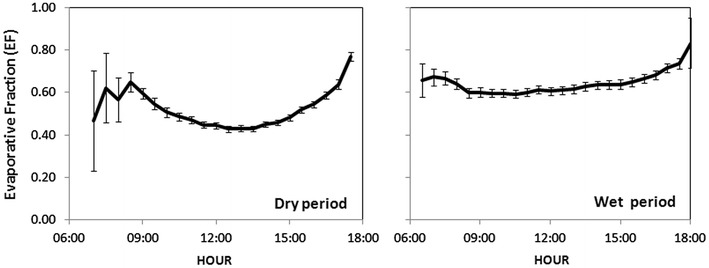
Daytime evolution of the half-hour mean evaporative fraction (EF) for both the wet and dry periods. *Error bars* represent 95 % confidence interval

The EF daytime evolution presented in Fig. 8 shows a typical concave up shape which is more pronounced during the dry season than the wet one. During the dry season, the minimum daytime EF values were observed around noontime, and the highest values were found both in the early morning and late afternoon. During the wet season EF decreases rapidly from 0.8 at sunrise and reaches its lowest value of 0.6 at 8.30 a.m. This is probably due to the radiation increase that favors the water evaporation from the ecosystem. After that, EF remains relatively constant until 3.00 p.m. when the VPD reaches its highest value. These EF diurnal evolutions suggest that during the morning when the radiation increases (Fig. 6) with low VPD (Fig. 7), the sum of turbulent fluxes (H + LE) increases faster than the latent heat flux (LE) alone. An opposite EF behavior was observed in the afternoon with the decrease in radiation evolution and high VPD, i.e. the latent heat flux (LE) increases faster than the turbulent fluxes sum (LE + H). The diurnal cycles of H and LE reported in Africa for similar Sahelian [24, 60, 61] and Sudanian sites [22, 33, 62] support well this diurnal EF behavior. Overall, daytime values of EF ranged from 0.4 to 0.8 for the ecosystem investigated here. However, this overall diurnal EF behaviors, which has been hypothesized by Lhomme and Elguero [63] was also reported with consistent magnitudes for similar African sites using the eddy-covariance measurements, notably in Benin for cultivated savanna [30] and in Morocco for sparse vegetation [64, 65].

### Seasonal variations patterns of EF and WUE

**Fig. 9:**
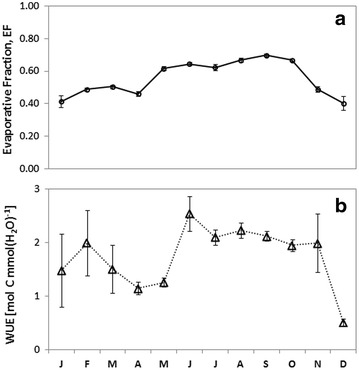
Seasonal evolution of the monthly average evaporative fraction (EF) and water use efficiency (WUE) during the whole measurements period. *Error bars* represent 95 % confidence interval

The EF and WUE seasonal variations (monthly mean) during the year are given in Fig. 9. As expected, similar contrasted trends were observed in WUE and EF seasonal variations. This was mainly due to variations of the vegetation growth and available energy in relation with main environmental conditions changes between the dry and wet seasons. During the dry season, WUE and EF, increased gradually from December due probably to the renewing leaves, reached overall in February and decreased to together with the air humidity increase, although the precipitation did not start yet [31, 35, 36]. This led to a significant increase in the water vapour flux from February at the studied site region [22, 30], and induced a WUE decrease from February to April despite the net CO_2_ absorption by a few perennial herbs and sparse trees keeping their green leaves or renewing them [37]. From May when the rain events became more frequent, EF and WUE increased progressively again and reached their maximum yearly values in August–September. From October, a decline trend was observed until December due to the significant reduction of the vegetation density and activity induced by crops harvest, frequent fires, senescence and desiccation processes during the drought, but also to the soil dryness inducing an evapotranspiration decrease. However, most of shrubs or trees keep some green leaves or renew them, and can satisfy their atmospheric demand of the water through their roots system which was able to use water from the deep layer. This might explain well the low, but still significant values of EF and WUE that were observed in the dry period.

Finally, monthly average values of WUE varied from 0.51 ± 0.07 to 2.54 ± 0.32 mmol C (mol H_2_O)^−1^ and EF from 0.40 ± 0.17 to 0.70 ± 0.01 (Fig. 9). Similar seasonal WUE trends were reported for similar African sites: in Benin for savanna [37] and forest (Ago et al. submitted); in Niger for savanna and millet crops [56, 57, 60]; and in Southern Africa for forest, woodland, shrubland and savanna [44, 58, 59].

As WUE, EF seasonal values and trends were also consistent with findings reported in Africa by several authors: in Benin for forest and savanna [22, 33] and in Kenya for woodland and grassland [66].

### Influence of rain events on ecosystem respiration R

**Fig. 10:**
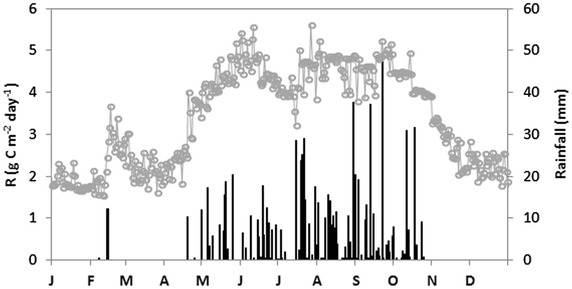
Influence of the rain events on the ecosystem respiration (R) during the year 2006. Data are the daily cumulated R and rainfall

In order to better analyze the impact of precipitation on the total ecosystem respiration R, we have displayed in Fig. 10 the evolutions during 2006 of daily sums of respiration and rainfall. There is an immediate increase of R after the first significant rain events following drought periods. This was clearly observed on March 14, April 19 and July 15 with cumulated rainfall respectively for 12.3, 10.4 and 28.6 mm. When no rain was recorded during a long period, a decrease was observed in R. Reversely, when rainfall events became more regular, the ecosystem respiration increased continuously before reaching their highest and stable values (~4.9 g C m^−2^ day^−1^) in August–September. After the last significant rain, the ecosystem respiration tended to decrease back to low values (~2.4 g C m^−2^ day^−1^) at the end of rainy season and during the subsequent dry season. During drought periods, the soil micro-organisms activity was very low and the soil wetted by first rains induced bursts in the activity of soil micro-organisms.

This sudden increase observed in the ecosystem respiration following significant rainfall after a relatively long drought period was also reported by several authors for other water-limited ecosystems in Africa: in Burkina Faso for savanna [48], in Niger for millet and savanna [55] and in South Africa for savanna [43], and elsewhere in the world: in USA for grassland and savanna [42, 67, 68] and in China for a typical steppe [69]. Indeed, in water-limited sites, first rain events cause generally a great stimulation in the soil microbial activity after a relatively long drought period.

### Seasonal variations of carbon fluxes (NEE, R and G_p_)

**Fig. 11:**
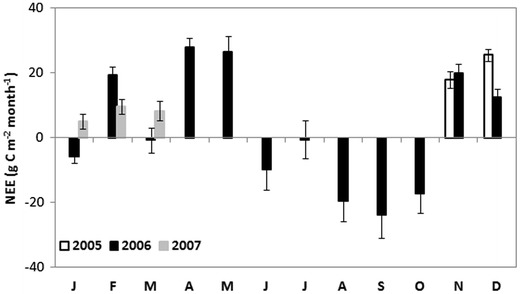
Evolution of monthly cumulated net ecosystem exchange (NEE) from 1 November 2005 to 31 March 2007. *Error bars* represent 95 % confidence interval

**Fig. 12:**
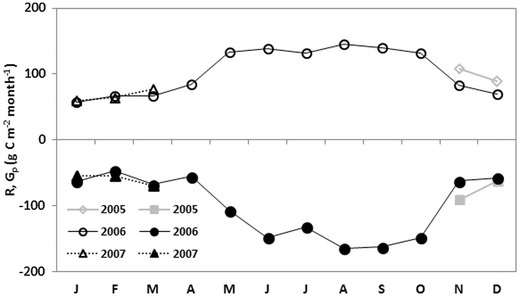
Evolution of the monthly cumulated ecosystem respiration (R) (*empty triangle*, *square*, *circle*) and gross primary productivity (G_p_) (*full triangle*, *square*, *circle*) from 1 November 2005 to 31 March 2007. *Error bars* represent 95 % confidence interval

In order to better understand the seasonal evolution of fluxes taking into account the changes observed in footprint areas between dry and wet seasons, monthly cumulated values of carbon fluxes (NEE, R and G_p_) are presented in Figs. 11 and 12. Overall, along the year, the carbon flux dynamics were unsurprisingly submitted to a strong seasonal variability. The site behaved as a carbon sink during the wet period while it was a carbon source during the drought and dry-to-wet transitional periods (Fig. 11). This suggests that G_p_ was consistently higher than R only during the wet periods when the vegetation greatly grew to reach its highest cover area at the site. The NEE evolution was likely related to that of both respiration R and photosynthesis G_p_.

During the dry season, G_p_ was significantly reduced (in absolute value) in comparison to their values during the wet season. The reduced G_p_ values during the dry season results mainly from the reduced density of green leaves which were still kept or renewed during this period by shrubs or trees, a few perennial herbs and late crops still non harvested which composed the ecosystem. G_p_ started to increase (in absolute value) after the first rain events in March 2006 when most of the green leaves started also their growth. A break in precipitation, as observed in April 2006 induced a drought with subsequent decay in the new initiated leaves, leading to a decrease in G_p_ (in absolute value). From May, when precipitations become more regular inducing probably an increase and stable soil moisture, especially from April to October, G_p_ increased and reached its maximum values in August–September. Therefore, the vegetation grew rapidly with a continuous increasing in the photosynthetic capacity. This was confirmed by the evolution of LAI from April to October. After the last rain events, especially during November–December, G_p_ tended to decrease to its lowest value due not only to the starting drought conditions (decrease in soil moisture and increase in VPD), but also to crop harvest, leaf senescence and desiccation processes.

Ecosystem respiration R also showed a high seasonal variation related to precipitation regime and seasonal vegetation development. During dry season, R was low probably due to the soil drought conditions induced by the lack of rain and vegetation growth reduction. While G_p_ (in absolute value) drops if first rains are not followed during the time by others, R increases from March to April. This suggests that the increase observed in soil water availability induced by first rains, appears sufficient to continue to stimulate the carbon release. During November–December. As for G_p_, R was low probably due to the soil dryness as no rain was recorded and green leaves density reduction. This suggests that during the wet-to-dry transitional and dry periods, the ecosystem has lost continuously carbon via the soil and little plants respiration, but at low rate, mainly due to the soil dryness.

However, during the wet season, if a significant drought appears both R and Gp would be reduced. This was especially observed during July 2006 with a significant drought induced by a decrease in precipitation ranging from 18 to 8 mm from June 18 to July 3 2006, followed by 12 continuous days without rains (Fig. 1e).

Similar seasonal variations of R and G_p_ between dry and wet seasons were reported in other studies for similar ecosystems in West Africa, especially in Benin for savanna [37] and forest (Ago et al. submitted), in Burkina Faso for savanna [48], in Burkina Faso for crop/fallow in Ghana [46] and in Niger for fallow savanna [49]; and elsewhere in Africa, e.g. for semi-arid savannas in Botswana [70] and in South Africa [71], and for papyrus and cocoyam wetland in Uganda [72].

### Annual net carbon exchange and implications for management strategy

During the year 2006, the cumulated NEE was +29 ± 16 g C m^−2^ (±CI) indicating that the site was close to an equilibrium ecosystem. In the years preceding the flux measurements period, the Nangatchori site has been highly disturbed as underlined by other authors [73, 74]. These disturbances (mainly deforestation and degradation) would explain the carbon behavior of the studied site in 2006 [1, 3, 7, 20, 49]. However, flux data collected for 17 months didn’t allow us to better analyze the sensitivity of the ecosystem to disturbance. Until now, no management site strategy was defined by the forest office.

These results were comparable with those reported by several authors in Africa for similar sites on a yearly basis. In West Africa, some sites acted as net carbon sinks: cumulated NEE of −232 ± 27 (±CI) for a cultivated savanna [37] and −640 ± 20 g C m^−2^ (±CI) for a forest (Ago et al. submitted) in Benin, −32 g C m^−2^ for a fallow savanna in Niger [49], −239 g C m^−2^ for a semiarid savanna grassland in Senegal [53], −387 ± 23 g C m^−2^ (±std) for a shrub savanna in Ghana [46]. In contrast, other sites acted as net carbon sources: cumulated NEE of +128 ± 7 and +108 ± 6 g C m^−2^ (± std) for a short grassland savanna and fallow/cropland in Ghana, respectively [46]. In South Africa, Archibald et al. [71] reported an average cumulated NEE of +99 ± 35 g C m^−2^ (±CI) for a semi-arid Savanna on 2 years. This large variability in patterns of African sites could be explained by the vegetation types, management systems and disturbance degrees as reported by several authors [46, 48, 49, 71].

## Conclusion

This study has analyzed the eddy covariance fluxes measured between 1 November 2005 and 31 March 2007 over a degraded woodland located in northern Benin (West Africa). To our knowledge, this is the first long term eddy-covariance data set of carbon flux that is analyzed over woodlands in West Africa.

This study has showed seasonal contrasted variations in carbon fluxes (NEE, R, G_p_), evaporative fraction (EF) and water use efficiency (WUE) in relation to the alternation between dry and wet seasons. Fluxes responses to the main environmental variables changes were studied. At the day scale, fluxes were mainly controlled by radiation and to a lesser extent by the VPD. Nighttime fluxes were observed to be strongly influenced by air humidity, but seemed quite insensitive to temperature. While no clear VPD impact on EF was observed likely due to the roots system capacity of trees or shrubs to use the water from deep layers of the ground during the drought, a limited VPD impact on WUE was found. However, with respect to the use of water resources, the Nangatchori site appeared more efficient during morning than afternoon, and evaporated more water around the noontime than both at sunrise and sunset. At the seasonal scale, the rainfall and probably the soil moisture appeared to be the main factors controlling the carbon fluxes variability between the dry and wet seasons. According to water use, the investigated site evaporated more and was more efficient during the wet than dry season.

Finally, the Nangatchori site was near of the equilibrium with a cumulated NEE of +29 ± 16 g C m^−2^ during the year 2006. In order to better clarify this vegetation pattern in the context of the climate change, fluxes observations during several years will be necessary. The ecosystem remains highly disturbed annually by frequent bushfires, intensive agriculture activities, illegal trees logging and cattle grazing.

## Methods

### Site description

The study was focused on a site (9.65°N, 1.74°E, 432 m) located in the Nangatchori village, approximately 20 km south of the Nalohou site which has already been described and studied by other authors [30, 33, 37]. The site is a typical degraded woodland that has been highly disturbed by illegal cattle pasture, intensive agriculture activities, tree logging and bushfire in the past years [36, 73, 74]. Northern Benin is a part of the Sudanian climatic region. It is characterized by an average annual rainfall of 1254 mm (1950–2009 average), of which 90 % occurred between April and October [33].

The soil at the Nangatchori site is a Luvisol skeleta chromic (FAO classification) composed mainly of 5–13 % clay, 77–85 % sand and 7–9 % silt on the surface horizon (0–50 cm depth) and 28–32 % clay, 50–56 % sand and 12–18 % silt in the roots zone [75]. In this region, most of the original landscapes have been undergoing hydric erosion [76, 77] for several decades. The site was composed by some sparse woody and herb vegetation, typical of the Sudanian region, with a species composition varying between dry and wet seasons [78, 79]. A floristic inventory during wet seasons of the most abundant species showed a heterogeneous vegetation composed mainly by some natural woody species (*Isoberlinia spp, Monotes kerstingii, Parkia* *biglobosa, Uapaca togoensis, Vitellaria* *paradoxa*), tree crops (*Blighia sapida, Anacardium occidentale, Tectona grandis*), annual crops (*Dioscorea spp, Manihot esculenta, Zea mays, Pennisetum spp,* *Sorghum bicolor*, *Arachis hypogaea*) and herbs (*Andropogon guayanus, Imperata cylindrical, Andropogon tectorum, Panicum maximum*). On the basis of LANDSAT TM scenes obtained in 2010, the area of 1 km^2^ around the flux tower was characterized. It was mainly constituted by the degraded woodland (28 %) and crops/fallows/bare soil (72 %) (Fig. 2). The main wind directions were South-West and North-East during the wet and dry seasons, respectively [36]. Generally, from November to March, most of herbaceous strata were burned by farmers with cultivated parcels mainly covered by crops residues and litter. Most of the leaves fall from trees occurred at the end of the wet season (October), except species such as *Isoberlinia spp* and fruit trees which kept their leaves during the dry season [28, 33, 37, 38].

### Fluxes, meteorology and other variables measured

Fluxes of CO_2_, water vapor and sensible heat were measured continuously at the Nangatchori village over a degraded woodland by the eddy covariance technique. The flux system, placed at 8 m above ground, approximately and respectively for 3 and 7 m above trees and crops canopies, was made up by a 3D sonic anemometer (Model Solent Research R3, Gill Instruments, Lymington, UK) coupled with an open-path infrared gas analyzer (IRGA, Licor 7500, Inc., Lincoln, NE, USA) that recorded the fluxes at a 8 Hz frequency.

The deployed meteorological station failed to measure complementary meteorological data at the site. As a back up solution, we have used the half-hour data recorded to the nearby site of Nalohou during the same period of the fluxes measurement. To do this, we have checked that the seasonal (daily) variability and magnitudes of the main meteorological parameters (i.e. mainly the radiation, temperature, vapour pressure deficit) were similar at a few kilometres scale in the whole Djougou district, except rainfall and soil moisture [22]. Details on measurements and calculations of main meteorological variables can be found in Ago et al. [37]. All these variables were sampled every 30 s and averaged half-hourly. The radiation sensors were calibrated by a comparison annually with standard sensors (CGR4 and CMP21 Kipp and Zonen, Delft, The Netherlands). Both fluxes and meteorological conditions were recorded for 7 months from 1st November of 2005 to 31th March of 2007.

The net carbon exchange (NEE) was computed every half hour as the algebraic sum of turbulent fluxes measured by eddy-covariance technique and of storage fluxes considering as negligible the additional terms [80]. As we did not have CO_2_ profile measurements throughout the canopy, storage flux term was computed from the single CO_2_ concentration measurements at 8 m above ground [37, 46, 51, 53, 61]. This approach can be generally criticized, but may be acceptable in the present study because of the openness of the canopy and relative low height of the eddy-covariance system [81].

CO_2_ fluxes calculation was performed using the Eddyflux Software package [82] and following the standard methodology proposed by Aubinet et al. [52]. The flux data treatment included the despiking, double rotation, spectral corrections and WPL correction. Fluxes of CO_2_ were submitted to a stationarity test [83] and only the data that met the quality test with a deviation lower than 60 % were used to establish the fluxes responses to driving meteorological factors. A photosynthetic photon flux density (Q_p_) criterion was used to separate data between night and day, with a threshold of 5 µmol m^−2^ s^−1^ [84]. In order to correct the nighttime fluxes error, a u*-filtering was applied [47, 52]. Like studies conducted in other African sites, the filtering criterion was chosen by a visual approach [37, 55, 71] and the u* threshold of 0.10 m s^−1^ was found for the investigated site. Data gaps in the flux time series resulting from the eddy-covariance system failures, power cuts or data removal because of poor quality or stable conditions were filled using the flux responses parameterizations to the main meteorological driving factors. For the site, 47 and 39 % of the missing data were filled respectively for the nighttime and daytime fluxes. For daytime gaps, we have used the Misterlich equation, Eq. (1) to describe the fluxes responses to radiation, i.e. Q_p_ [37, 80, 85]. This was done using 30-min data for 15 days windows.

1 $${\text{NEE}} = - \left[ {A_{max} + r_{d} } \right]*\left[ {1 - exp\left\{ {\frac{{ - \alpha Q_{p} }}{{\left( {A_{max} + r_{d} } \right)}}} \right\}} \right] + r_{d}$$

where ${\text{NEE}}$ is the net ecosystem exchange, $Q_{p}$ the photosynthetic photon flux density, $r_{d}$ the dark respiration; $\alpha$ the quantum light efficiency and $A_{max}$ the ${\text{NEE}}$ at the light saturation.

A non-linear regression was performed to deduce the three characteristic parameters using the Levenberg–Marquardt algorithm (MATLAB, R2010b version, The Mathworks, Natick, USA).

The site is characterized by a low seasonal (daily mean) variability in temperature (<10 °C) and large changes in precipitation often accompanied with variation of both the atmospheric humidity and soil moisture. In Sudano-Sahelian climate, these two variables (temperature and soil moisture) co-vary generally at the seasonal scale [22, 33, 53, 86]. Therefore, the nighttime fluxes were not predicted to vary significantly with temperature [39, 41] as in other similar African sites [37, 43, 44]. Nighttime fluxes would depend strongly on soil moisture. As no soil moisture data was recorded for the site, the average of nighttime fluxes ${\text{r}}_{\text{n}}$ unavailable were filled by the exponential relationship between $r_{n}$ (u* > 0.10 m s^−1^) and the nighttime average of relative humidity ($RH_{n}$), i.e. Eq. (2) following:

2 $$r_{n} \;{ = }\;{\text{a*Exp }}({\text{b*}}\;RH_{n} )$$

where $a$ is the minimum value of the nighttime fluxes average and $b$ a parameter characterizing the sensitivity of the average nighttime respiration to RH. These two parameters ($a$ and $b$) were determined using the whole nighttime fluxes of unstable conditions (u* > 0.1 m s^−1^) during the whole period covered by the eddy-covariance measurements. The daily sum of the nighttime ecosystem respiration $(R_{n} )$ was estimated by $R_{n} = r_{n} *DL$, with $DL$ (s day^−1^) representing the night length of a day.

Flux-partitioning was performed into two main components, i.e. gross primary productivity ($G_{p}$) and ecosystem respiration $({\text{R}})$. In order to better take into account the photosynthesis processes occurring during day conditions, we have estimated the daily sum of daytime ecosystem respiration $(R_{d} )$ using the dark respiration, r_d_, provided by the light-responses of the daytime flux measurements from Eq. (1) above by $R_{d} = r_{d} *DL$, where $DL$ (s day^−1^) is always the light length of a day. This approach has been recently more used to derive the daytime respiration by several authors [84, 87–90]. It can complete the methods based on the nighttime flux [52, 91] using the nighttime respiration responses to the main meteorological variables such as temperature [46, 48, 92] or soil moisture [37]. Thus, $G_{p}$ was inferred by subtracting the total daily sum of ecosystem respiration ${\text{R}}$ from ${\text{NEE}}$ calculated from half-hourly measured fluxes by the eddy-covariance technique or gap-filled. The daily sum of R was obtained by adding $R_{d}$ and $R_{n}$.

The sampling error on individual gap-filled flux was estimated for both daytime and nighttime fluxes to twice the standard error (95 % confidence interval) of the gap-filling model residuals [37, 93]. Finally, the total uncertainty on flux (${\text{NEE}}$) was computed (95 % confidence interval) using the Richardson and Hollinger [94] approach assuming that the two error sources on its two major components were independent.

As the site is composed by a heterogeneous vegetation cover, we have made a footprint analysis during the unstable and neutral conditions in order to determine the contribution of each source to the measured fluxes. This was done for the whole measurements period for the two major wind directions using the software tool proposed by Neftel et al. [95]. This method is based on the two-dimensional analytical footprints model according to the Kormann–Meixner footprint model [96]. This model was applied to each half-hour value of the flux to obtain the contribution of each vegetation type to the measured fluxes during the wet and dry seasons. The model inputs are the main variables that are supplied by the eddy-covariance system i.e., the measurement height, friction velocity, displacement height, Obukhov length, horizontal wind velocity, standard deviation of the cross wind speed and wind direction.

Water use efficiency (WUE) is defined as the ratio of absolute values of ${\text{NEE}}$ and water vapour flux $E$ [55, 57, 97]. It was calculated using only ${\text{NEE}}$ values corresponding to $Q_{p}$ ≥ 400 µmol m^−2^ s^−1^ [37], as it is found to be sensitive to lower radiation when no water stress is present [98].

Leaf Area Index (LAI) time series were obtained by a combination of satellites LAI products (SEVIRI) constrained by in situ measurements. Main species dominating the ecosystem were inventoried in a 1 km^2^ plot surrounding the tower during wet period when the vegetation was fully developed.

Finally, the evaporative fraction, EF (%) was calculated following Eq. (3) below in order to analyze the fraction of the available energy, i.e. the sum of latent (LE) and sensible heat (H) fluxes which is converted into evapotranspiration:

3 $$EF = \frac{LE}{LE + H}$$

## Abbreviations

AMMA: African Monsoon Multidisciplinary Analyses
RH: relative humidity
K: light index
LAI: Leaf Area Index
H: sensible heat
LE: latent heat
Q_p_: photosynthetic photon flux density
VPD: vapour pressure deficit
NEE: net exchange ecosystem
WUE: water use efficiency
EF: evaporative fraction
R: ecosystem respiration
G_p_: gross primary productivity
NA: November–April
AM: April–May
MO: May–October
ON: October–November
CI: 95 % confidence interval
Std: standard deviation
